# Antihyperglycemic Effects of *Annona cherimola* Miller and the Flavonoid Rutin in Combination with Oral Antidiabetic Drugs on Streptozocin-Induced Diabetic Mice

**DOI:** 10.3390/ph16010112

**Published:** 2023-01-12

**Authors:** Miguel Valdes, Fernando Calzada, Jesús Martínez-Solís, Julita Martínez-Rodríguez

**Affiliations:** 1 Instituto Politécnico Nacional, Sección de Estudios de Posgrado e Investigación, Escuela Superior de Medicina, Plan de San Luis y Salvador Díaz Mirón S/N, Col. Casco de Santo Tomás, Miguel Hidalgo, Mexico City CP 11340, Mexico; 2 Unidad de Investigación Médica en Farmacología, UMAE Hospital de Especialidades 2° Piso CORSE Centro Médico Nacional Siglo XXI, Instituto Mexicano del Seguro Social, Av. Cuauhtémoc 330, Col. Doctores, Mexico City CP 06720, Mexico

**Keywords:** *Annona cherimolla* Miller, diabetes mellitus, flavonoids, rutin, oral antidiabetic drugs

## Abstract

Ethanolic extract obtained from *Annona cherimola* Miller (EEAc) and the flavonoid rutin (Rut) were evaluated in this study to determine their antihyperglycemic content, % HbA1c reduction, and antihyperlipidemic activities. Both treatments were evaluated separately and in combination with the oral antidiabetic drugs (OADs) acarbose (Aca), metformin (Met), glibenclamide (Gli), and canagliflozin (Cana) in acute and subchronic assays. The evaluation of the acute assay showed that EEAc and Rut administered separately significantly reduce hyperglycemia in a manner similar to OADs and help to reduce % HbA1c and hyperlipidemia in the subchronic assay. The combination of EEAc + Met showed the best activity by reducing the hyperglycemia content, % HbA1c, Chol, HDL-c, and LDL-c. Rutin in combination with OADs used in all treatments significantly reduced the hyperglycemia content that is reflected in the reduction in % HbA1c. In relation to the lipid profiles, all combinate treatments helped to avoid an increase in the measured parameters. The results show the importance of evaluating the activity of herbal remedies in combination with drugs to determine their activities and possible side effects. Moreover, the combination of rutin with antidiabetic drugs presented considerable activity, and this is the first step for the development of novel DM treatments.

## 1. Introduction

Diabetes Mellitus (DM) is a chronic disease characterized by high blood glucose levels as a consequence of resistance or lack of insulin secretion from the pancreas [1]. This disease affected 463 million people worldwide in 2019 [2]. DM caused 1.6 million deaths in 2016 and is considered as one of the main causes of deaths globally [3]. DM type 2 accounts for 90 to 95% of all diabetes cases and its specific etiology remains unknown [4].

The pathophysiology of DM type 2 consists of a heterogeneous field where progression can considerably vary. The changes occurring in the metabolism of carbohydrates and proteins are a consequence of several disturbances defined as ominous octets [5]. The excess quantity of glucose in the blood stream causes micro- and macrovascular damage as a result of the increase in free radicals [6] that react with tissue proteins via a non-enzymatic oxidation process in the hemoglobin and explains the synthesis of many advanced glycation end-products (AGEs) as glycated hemoglobin (HbA1c) [7]. HbA1c values reflect the average values of fasting blood glucose levels [8] and are used as parameters to continuously monitor patients with this disease [9,10]. Moreover, hyperglycemia is related to the changes occurring in the lipid metabolism process characterized by hypertriglyceridemia, high levels of cholesterol, and the alteration between low-density (LDLs) and high-density (HDLs) lipoproteins [11] that are associated with an increase in mortality rates.

The management of DM is a standing problem that requires a continued search of the possible alternatives. Numerous attempts have been made to address this situation and, at present, there several oral antidiabetic drugs exist [12], which are classified according to their mechanism of action into secretagogues, such as glibenclamide, which joins the cell receptor SUR1 and causes insulin release. Sensitizers, such as metformin, which improve the consumption rate of glucose, and antihyperglycemics, such as acarbose or canagliflozin, which can avoid the absorption of glucose or stimulation of urinary glucose excretion, thus reducing high blood glucose levels, have also been considered in the literature. However, the goal of glycemic control has not yet been achieved in the research due to the limitations induced by their use for a long period of time [13,14]. In this sense, the search for more effective treatments is urgent [15]. Medicinal plants are the main ingredients that have been used in traditional medicine [16]; in this sense, medicinal plants can be used to complement treatments to achieve improved glycemia control. *Annona* genera include several species that have presented antidiabetic properties [17,18]. *Annona cherimola* Miller (*A. cherimola*) is a fruit tree that belongs to the annonaceae family [19] and is a perennial species showing the presence of its leaves almost all year round, and it is widely cultivated and distributed in subtropical areas around the world [20], mostly for its fruit known as “annona” or “cherimoya” [20,21]. Local populations also use its leaves as a remedy to treat several illnesses, such as gastrointestinal disorders, worms, and diarrhea [22]. Studies have proved that the leaves of this species have antidepressant [23] and pro-apoptotic [24] activities. In respect to the toxicity of *Annona cherimola*, it has been evaluated according to the OCDE procedure in a mouse model, and the results presented a median lethal dose (LD_50_) higher than 3000 mg/kg; these results suggest that it is safe to use [25].

All the compounds present in the leaves of *Annona cherimola* have recently been listed in the literature [26]. Flavonoids are highlighted due to their wide-ranging properties [27]. Moreover, previous studies indicated the presence of antihyperglycemic components in polar fractions [28], such as the flavonoid rutin, one of the principal secondary metabolites responsible for the antihyperglycemic activity of *A. cherimola*. Rutin is present in higher quantities compared to other flavonoids in leaf extracts and exerts antioxidant effects and lowers glucose diffusion properties [29]. This effect can be enhanced by its structure that provides it with α-glucosidase inhibitory activity [30], as well as other activities [31], whose mechanisms are already known in the field [32] and contribute to the antihyperlipidemic activity [33] of this species. Although the antihyperglycemic effect of ethanolic extract obtained from *A. cherimola* and rutin administrated alone has already been studied, there are no acute or subchronic studies that evaluate their combination with oral antidiabetic agents. Thus, the aim of the present study is to assess the effect of *A. cherimola* ethanolic extract and the flavonoid rutin administered alone and in combination with oral antidiabetic drugs on hyperglycemic states in acute and subchronic studies in animals and its effects on other parameters, such as HbA1c and lipid profiles.

## 2. Results

### 2.1. Acute Antihyperglycemic Effects of Ethanolic Extract from Annona cherimola, Rutin, and Oral Antidiabetic Drugs on SIT2D Mice Model

First, the acute effect of ethanolic extract obtained from *Annona cherimola* Miller (EEAc), rutin, and oral antidiabetic drugs (OADs) were measured in SIT2D mice following the administration of treatments separately. We observed that the groups treated with EEAc or glibenclamide showed a significant decrease in blood glucose levels at 2 h of treatment, the group treated with the flavonoid rutin presented a significant decrease at 4 h of treatment, and the group treated with canagliflozin showed a significant decrease at 2 and 4 h of treatment (Table 1).

When the OADs were combined with the EEAc, all treatments presented better effects on the hyperglycemia rather than separately; all combinations with EEAc showed a significant decrease at 2 and 4 h of treatment (Table 1).

On the other hand, when the OADs were combined with rutin, the combination of rutin + acarbosa and rutin + glibenclamide showed a significant decrease at 4 h of treatment; in the case of the treatment of rutin + canagliflozin, a significant decrease was presented at 2 and 4 h of treatment (Table 1).

### 2.2. Subchronic Effects of Ethanolic Extract from Annona cherimola, Rutin, and Oral Antidiabetic Drugs on SIT2D Mice Model

Once we demonstrated the acute activity over hyperglycemia of the treatments alone or in combination, we decided to perform a chronic evaluation for 8 weeks.

**Figure 1:**
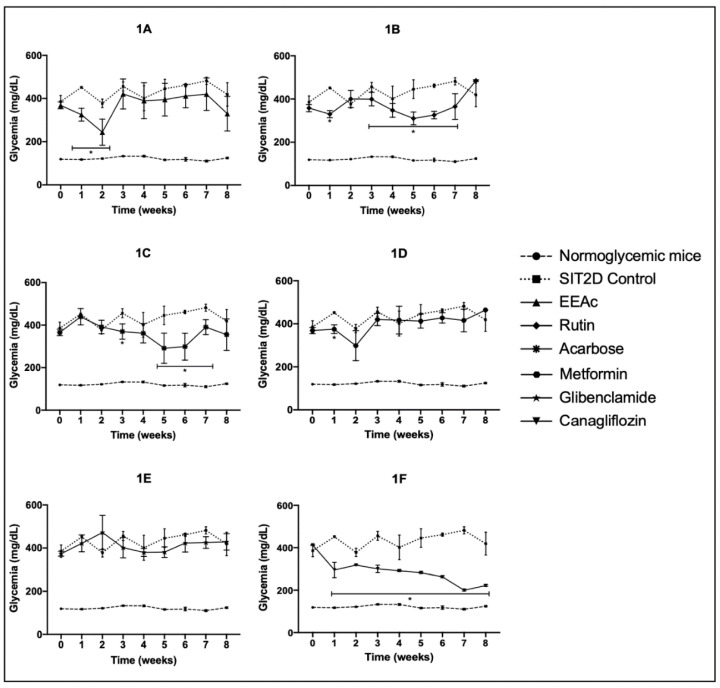
Effect over glycemic values of separate administration of the treatments in SIT2D mice compared with SIT2D and normoglycemic control groups. Groups treated with EEAc 300 mg/kg (**1A**), rutin 50 mg/kg (**1B**), acarbose 50 mg/kg (**1C**), metformin 850 mg/kg (**1D**), glibenclamide 5 mg/kg (**1E**), and canagliflozin 50 mg/kg (**1F**). Results are expressed as the mean ± SEM, *n* = 6, * *p* < 0.05 vs. SIT2D control at same week of treatment.

Following the chronic administration of the treatments, we observed that the group treated with EEAc showed a significant reduction in hyperglycemic values at weeks 1 and 2; however, the animals returned to their previous hyperglycemic values in week 3 and these values were maintained, similar to the SIT2D control, for the rest of the treatment (Figure 1A). The group treated with rutin showed a significant reduction in hyperglycemic values at weeks 3 to 7 (Figure 1B). In the case of the OADs, the group treated with acarbose presented a significant reduction in hyperglycemic values in weeks 3 and 5 to 7; however, during week 8 of the treatment, the glycemic values increased, reaching SIT2D control values (Figure 1C). The group treated with metformin presented a significant reduction in its hyperglycemic values in week 1 only; during the rest of the weeks, glycemic values similar to the SIT2D control were presented (Figure 1D). The group treated with glibenclamide did not present significant activity exceeding the hyperglycemic values (Figure 1E). The group treated with canagliflozin presented a significant reduction in hyperglycemic values from the first week until the end of the treatment (Figure 1F).

**Figure 2:**
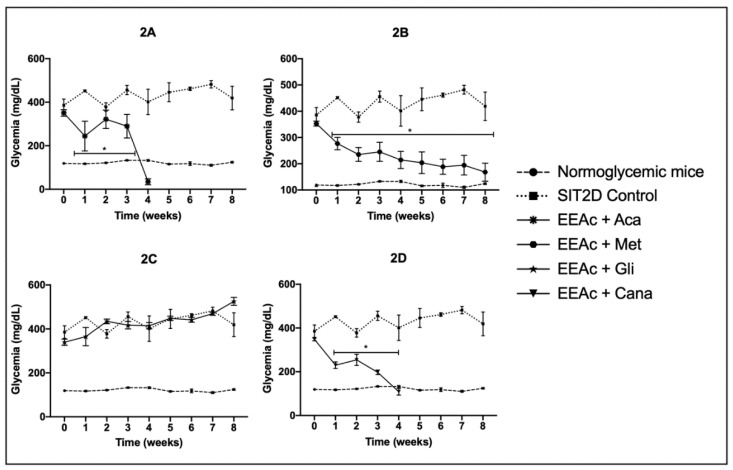
Effect over glycemic values of combined administration of the oral antidiabetic drugs with EEAc in SIT2D mice compared with SIT2D and normoglycemic control groups. Groups treated with EEAc 300 mg/kg + acarbose 50 mg/kg (**2A**), EEAc 300 mg/kg + metformin 850 mg/kg (**2B**), EEAc 300 mg/kg + glibenclamide 5 mg/kg (**2C**), EEAc 300 mg/kg + canagliflozin 50 mg/kg (**2D**). Results are expressed as the mean ± SEM, *n* = 6, * *p* < 0.05 vs. SIT2D control at same week of treatment.

When the OADs were chronically administered in combination with the EEAc, we observed that EEAc + acarbose reduced hyperglycemia from the first week; however, in week 4, we observed the death of all the treated animals (Figure 2A) and the mean value of the blood glucose levels for the dead animals was 43.5 ± 13.8 mg/dL. The group treated with EEAc + metformin presented a significant reduction in its hyperglycemic values from the first week and this was maintained until the end of the treatment, close to the normoglycemic values (Figure 2B). The group treated with EEAc + glibenclamide did not present a reduction in its blood glucose levels (Figure 2C); finally, the group treated with EEAc + canagliflozin presented a significant reduction in its hyperglycemic values from the first week; however, at week 4, we observed the death of all the animals treated with this combination (Figure 2D)—the mean value of the blood glucose levels for the dead animals was 100.5 ± 15.1 mg/dL.

**Figure 3:**
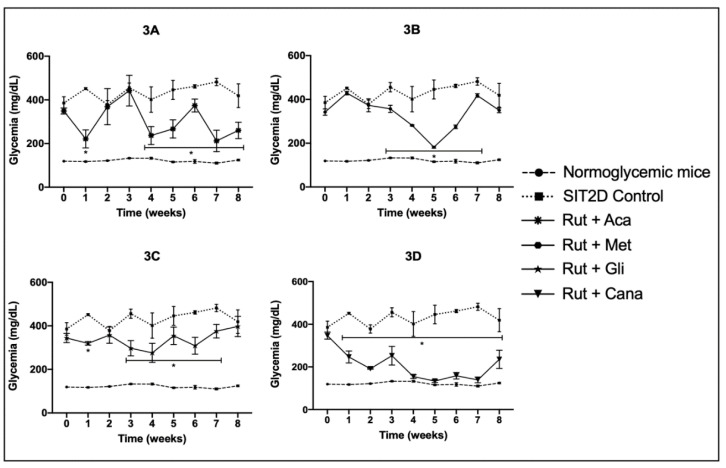
Effect over glycemic values of combined administration of the oral antidiabetic drugs with rutin in SIT2D mice compared with SIT2D and normoglycemic control groups. Groups treated with rutin 50 mg/kg + acarbose 50 mg/kg (**3A**), rutin 50 mg/kg + metformin 850 mg/kg (**3B**), rutin 50 mg/kg + glibenclamide 5 mg/kg (**3C**), rutin 50 mg/kg + canagliflozin 50 mg/kg (**3D**). Results are expressed as the mean ± SEM, *n* = 6, * *p* < 0.05 vs. SIT2D control at same week of treatment.

When the OADs were chronically administered in combination with rutin, we observed that the group treated with rutin + acarbose presented a significant reduction in its hyperglycemic values during the first week of treatment and during the fourth week until the end of the treatment (Figure 3A). The group treated with rutin + metformin presented a significant reduction in its hyperglycemic values from the third until the seventh week; the glycemic values began to increase until achieving hyperglycemic values similar to the SIT2D control (Figure 3B). The group treated with rutin + glibenclamide showed a significant decrease in its blood glucose levels in the third until the seventh week (Figure 3C). In the case of the group treated with rutin + canagliflozin, this treatment generated a significant reduction in the hyperglycemic values from the first week until the end of the treatment, attaining values almost similar to the normoglycemic values obtained during the fourth week of treatment (Figure 3D).

### 2.3. Effects over Glycated Hemoglobin Levels after Chronical Administration of Ethanolic Extract from Annona cherimola, Rutin, and Oral Antidiabetic Drugs on SIT2D Mice Model

**Figure 4:**
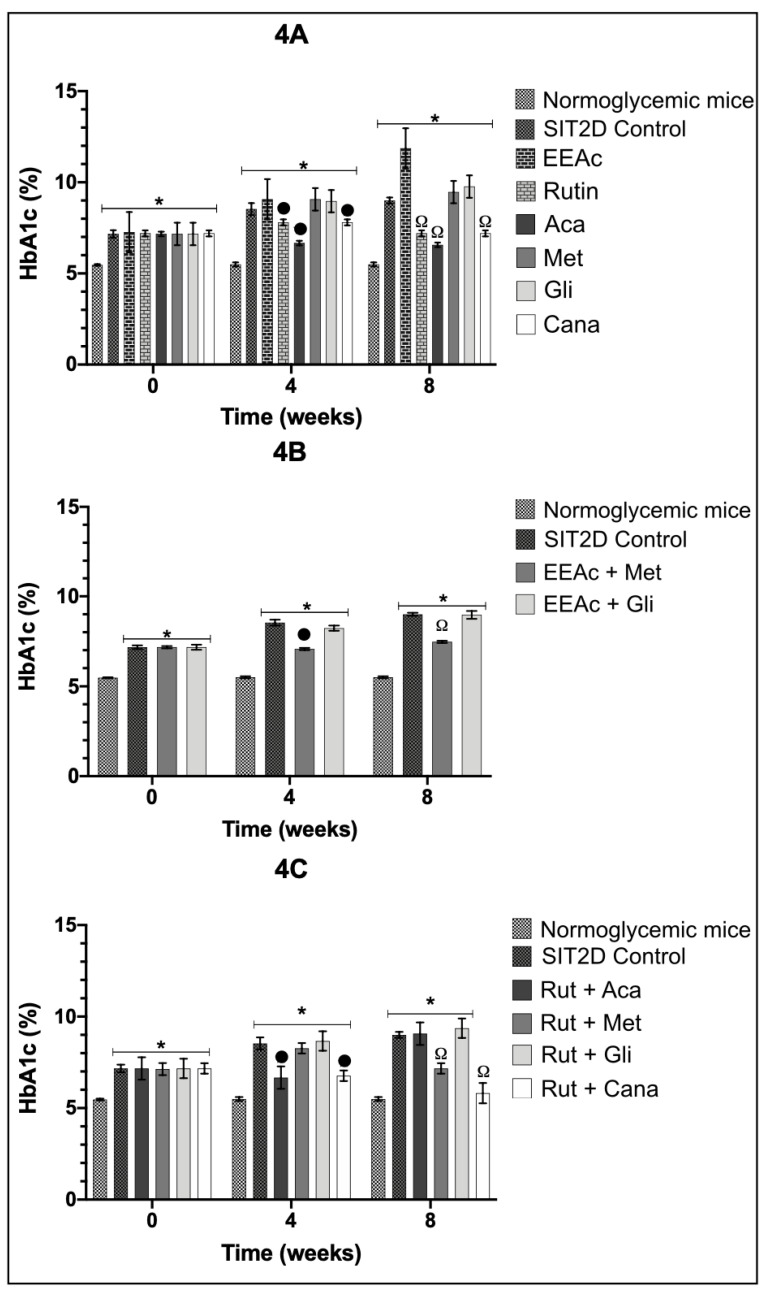
Effect over % glycated hemoglobin (% HbA1c), values after administration of the treatments separately and in combination. Groups administrated with treatments separately (**4A**), groups administrated with the combination of oral antidiabetic drugs and EEAc (**4B**), groups administrated with the combination of oral antidiabetic drugs and rutin (**4C**). Results are expressed as the mean ± SEM, *n* = 6, * *p* < 0.05 vs. Normoglycemic mice at same week of treatment; • *p* < 0.05 vs. SIT2D control in week 4; Ω *p* < 0.05 vs. SIT2D in week 8.

The subchronic administration of the individual treatments generated a significant decrease in the percentage of glycated hemoglobin (% HbA1c) at weeks four and eight in the groups treated with rutin, acarbose, and canaglifozin, in comparison with the SIT2D group (Figure 4A), when the OADs were administered in combination with EEAc. The combination of EEAc + acarbose and EEAc + canagliflozin caused the death of the animals in week four, due to the fact that % HbA1c could not be measured in both groups. The administration of the combination of EEAc + glibenclamide did not generate a significant reduction in % HbA1c (Figure 4B).

In the case of the combination of OADs with rutin, we observed a significant reduction in % HbA1c following the administration of rutin + acarbose in weeks four and eight. In the case of rutin + canalgiflozin, a significant reduction in the % of HbA1c was observed at weeks four and eight, achieving normoglycemic % HbA1c values in week eight. It is important to mention that the groups treated with rutin in combination with OADs did not kill the animals when compared to the mortality rates resulting from the combinations of EEAc + Aca and EEAc + Cana. On the other hand, for the groups treated with rutin + metformin and rutin + glibenclamide, we did not observe a significant reduction in % HbA1c (Figure 4C) during the administration of the treatments.

### 2.4. Effects over Lipid Profile Levels after Chronical Administration of Ethanolic Extract from Annona cherimola, Rutin, and Oral Antidiabetic Drugs on SIT2D Mice Model

At the end of the treatment, lipid profiles were measured for all the study groups. We observed that the SIT2D control group significantly increased the values of Chol, Tri, and LDL-c in comparison with the NM control. In the case of HDL-c, these values were significantly reduced in comparison with the NM control and the atherogenic index plasma (AIP); this index was significantly increased in comparison with the NM control index. In the case of the treatments, we observed that the groups treated with EEAc, rutin, and canagliflozin presented lower values with significant differences, in comparison with the SIT2D control group, these values being similar to the NM control group. The group treated with acarbose presented a significant increase in Tri values in comparison with the SIT2D and NM control groups; the remaining parameters were similar to the NM control group. In the case of the groups treated with metformin and glibenclamide, they did not show a difference in comparison to the SIT2D group; moreover, the AIP calculated showed a significant increase in comparison with the AIP of the NM control group (Table 2).

The subchronic administration of OADs in combination with EEAc resulted in the death of the animals treated with EEAc + Aca and EEAc + Cana; thus, the lipid profile could not be measured. In the case of EEAc + Met, the parameters Chol, HDL-c, and LDL-c were maintained in a manner similar to the NM control group; however, the Tri values significantly increased in comparison to the NM and SIT2D controls. The group treated with EEAc + Gli presented similar behavior to the SIT2D control with a significant increase in the AIP in comparison to the NM control (Table 3). Finally, the combination of OADs with rutin showed that Rut + Aca, Rut + Gli, and Rut + Cana maintained the values of Chol, Tri, LDL-c, and AIP in a manner similar to the NM control and showed a significant increase in the HDL-c value in comparison to the NM control. The group treated with Rut + Met presented activity similar to the SIT2D control with a significant increase in the AIP value (Table 4).

## 3. Discussion

Diabetes mellitus (DM) is one of the most prevalent chronic diseases worldwide; thus, its management requires new alternatives that will help to control the hyperglycemia levels of patients with DM [1,3]. To date, a wide variety of drugs are used to control hyperglycemia; nevertheless, prolonged use can result in adverse effects [12]. One of the principal approaches towards the use of oral antidiabetic drugs (OADs) is to reduce hyperglycemia values; in this sense, the correct management of hyperglycemia can be reflected in the adequate good control of the percentage of glycated hemoglobin (% HbA1c); however, not all OADs have good control of this parameter. In the long term, the increase in % HbA1c is related to an elevated cardiovascular risk [4], such as diabetic nephropathy and glaucoma, among the other DM complications [34,35,36]. Furthermore, some of the most common diseases that accompany patients with DM are dyslipidemias; if they are not adequately controlled, they can generate vascular complications as well as acute myocardial infarctions that can cause the death of the patient [37]. In patients with DM, it has been demonstrated in the research that the administration of sulfonylureas as well as thiazolidinediones may help to reduce the values of hyperlipidemia [38]. In addition to % HbA1c, several treatments performed on patients with DM2 help to reduce dyslipidemias; however, in the long term, all of them did not adequately control this problem. As a result, it is important to search for new treatments that can help to reduce and control the complications implicated by DM.

The aim of this study was to determine the effects of hyperglycemia, % HbA1c, and hyperlipidemic values following the chronic administration of ethanolic extract obtained from the leaves of *A. cherimola* and the flavonoid rutin, a compound isolated from *A. cherimola* that has been demonstrated to be one of the metabolites present in the plant that is responsible, in part, for their antihyperglycemic activity [28], as well as OADs commonly used in therapy. All the treatments were first administered alone and the combination of antidiabetic drugs with the extract and antidiabetic drugs with rutin was used to evaluate their activity.

First, all the treatments were evaluated separately. It is important to mention that the doses evaluated in the study were selected according to the posology of every OAD; in the case of EEAc and rutin, both doses were selected as reported in previous studies [25]. The reduction in the hyperglycemia observed was consistent with the results reported by other authors [26,27,28]. In the case of the OADs, the values obtained following the administration of acarbose were similar to those obtained for the evaluation of rutin; this can be explained by the fact that both molecules share a similar action mechanism to reduce the hyperglycemia mediated by the inhibition of α–glucosidase enzymes [28]. Moreover, rutin is a glucoside conformed by quercetin linked to rutinoside disaccharide; this disaccharide is structurally similar to acarbose, and it has been described as helping the interaction with the a-glucosidase enzyme inhibiting it [25,28]. In the case of metformin and glibenclamide, they presented poor activity over the hyperglycemia values. Perhaps, in the case of metformin, a daily administration is needed to achieve good control of hyperglycemia; in the case of glibenclamide, this can be explained due to the destruction of β cells following the administration of streptozocin to induce the SIT2D model [39,40]. Perhaps, there are not enough β cells to generate an adequate acute secretion of insulin to reduce the hyperglycemic values. In all cases, the combination of EEAc with antidiabetic drugs reduces hyperglycemia in the treated animals; it is possible that the pharmacological effect of each antidiabetic drug was favored as a synergism with some of the metabolites present in the EEAc. Several products have been isolated from *A. cherimola*, such as flavonoids, alkaloids, acetogenins, sterols, and sesquiterpenes, among others [17]; it is possible that the combination of antidiabetic drugs with one or more of the products present in the extract helps to reduce hyperglycemia in the treated animals.

Flavonoids have been widely reported in the literature as molecules presenting antihyperglycemic activity in vivo and in vitro [41,42,43]. Considering the abovementioned issues and that a bio-guided phytochemical study conducted by our research group led to the isolation of rutin as one of the products with antidiabetic effects present in *A. cherimola* [26,28], we decided to evaluate the activity of this flavonoid in combination with antidiabetic drugs. The combination of rutin with acarbose and canagliflozin was the treatment with the greatest reduction in the hyperglycemic values. We suggested that this was due to the combination of the mechanism of action. Rutin and acarbose have been commonly described as α-glucosidase inhibitors [28,44]; it is possible that the combination of these two compounds in the doses administered were enough to generate a greater reduction in the α-glucosidase enzyme. On the other hand, canagliflozin is a sodium glucose co-transporter-2 (SGLT2) inhibitor [45]; we suggested that the combination of rutin with a SGLT2 inhibitor, such as canagliflozin, may help to completely reduce the postprandial peak of glucose following food intake due to the combination of both mechanisms of action [46]. The reduction in the complex carbohydrates’ hydrolysis combined with the inhibition of glucose absorption may result in the significant reduction in hyperglycemia. The combination of rutin with glibenclamide and metformin did not generate a significant reduction in the hyperglycemic values in acute evaluation; however, we attributed this result to the necessary long-time administration of the treatments to achieve a reduction in the hyperglycemic values. Considering our results, the subchronic evaluations of all treatments previously described were performed with the aim to observe the effect of the hyperglycemic values on the long-time administration as well as the effect on the % HbA1c and hyperlipidemic values.

The subchronic evaluation of the separate treatments showed that the subchronic administration of EEAc, rutin, acarbose, and canagliflozin significantly reduced hyperglycemia in the treated animals; in the case of EEAc, the effect was maintained from weeks 1 to 4, and this was consistent with the activity demonstrated in previous subchronic studies [28]. However, after the fourth week, antihyperglycemic activity was lost; this may have been due to the dose of 300 mg/kg not being adequate to achieve normoglycemic values in a subchronic evaluation; thus, we propose the consideration of future experiments with the evaluation of EEAc at higher doses to observe the acute and chronic antihyperglycemic activities. The significant reduction in hyperglycemia following the administration of rutin is similar to that observed in the group treated with acarbose; this result is consistent with the result obtained in the acute evaluation of both treatments, and we attribute this result to the similar mechanism of action that has been described for rutin, an α-glucosidase inhibitor [28]. In the case of the group treated with canagliflozin, it achieved considerable control of the hyperglycemic values; we attribute this result to the mechanism of action of this drug, an SGLT2 inhibitor, and it has also been reported in the literature that monotherapy performed with canagliflozin lowers blood glucose levels independently from insulin and, with SGLT2 inhibition, reduces the renal glucose absorption of glucose and increases the excretion of glucose in the urine [45]. The previously noted effects on hyperglycemia were reflected in % HbA1c; in the case of EEAc, the inadequate diminution of hyperglycemic values at weeks 4 and 8 significantly increased % HbA1c in a manner similar to the SITD2 control. On the other hand, in the groups treated with rutin, acarbose, and canagliflozin, the reduction in the hyperglycemia values was reflected in the significant reduction in % HbA1c. We propose that the effective reduction in high blood glucose levels is related to the decrease in % HbA1c due to the fact that a high concentration of glucose in the bloodstream does not exist; thus, the reduction in glucose to form Schiff bases and Amadori products to produce advanced glycation end-products (AGEs) cannot be performed [47,48]. In respect to the lipid profile, EEAc, rutin, acarbose, and canagliflozin showed a significant reduction in Chol, Tri, and LDL-c with an increase in HDL-c values; in the cases of EEAc and rutin, our results are similar to those reported in the previous studies [26,49]. Studies of *A. cherimola* reported that subchronic treatment performed with an infusion from this plant helped to reduce cholesterol and triglycerides in diabetic mice and led an increase in HDL values, this activity being attributed to phenolic compounds [25]. In respect to rutin, it has been demonstrated in the literature that flavonoids can also reduce hepatic peroxidation and lead to a decrease in the synthesis of cholesterol. This is partly mediated by the regulation of fatty acid and cholesterol metabolism, affecting the gene expression of regulatory enzymes [25,50]. In respect to the atherogenic index plasma (AIP), the values calculated for the animals treated with EEAc, rutin, acarbose, and canagliflozin were similar to the NM control; we associated our results with these treatments possibly reducing the chance of increased cardiovascular risk, vascular damage, and mortality risk, which are some of the most common complications associated with secondary diabetes [4]; however, there is a need to conduct further experiments to confirm our hypothesis.

When the OADs were administered in combination with EEAc, we observed that the groups treated with acarbose and canagliflozin showed a reduction in hyperglycemia from the first week of treatment; however, all the animals died in week four. We discarded the possibility that the mortality observed after four weeks of treatment were possibly due to the toxicity generated for the administration of the extract since there are experiments that demonstrate the toxicity of the ethanolic extract obtained from the leaves of *A. cherimola*, categorized as number 5 in the safe or not label >2000 mg/kg [25]. According to our observations, we suggest that the death of the animals was due to the possible hypoglycemic activity after the repeated administration of both combinations; this could have been due to the quantity of drugs or EEAc administered in combination to the animals. We propose further experiments in the future to determine the correct doses to reduce hypoglycemia and to obtain normoglycemic values, avoiding the possible mortality rate increase as a result of the treatments. The group treated with the combination of EEAc + glibenclamide did not generate a significant reduction in hyperglycemic values; this could have been due to the principal effect of STZ to generate experimental diabetes mellitus in the β cells’ destruction [46]. In this sense, the molecular mechanism of glibenclamides is insulin secretion following the β cells’ stimulation [51]. In consideration of the abovementioned factors, if no β cells are present, the secretion of insulin and, consequently, the reduction in hyperglycemia cannot be achieved. In the case of the combination of EEAc + metformin, we observed a progressive reduction in the hyperglycemic values reaching the normoglycemic control group values; in comparison with the separate administration of metformin, the control of hyperglycemia was enhanced. We propose that the effect observed was due to EEAc generating a reduction in complex disaccharides mediated by the inhibition of the α-glucosidase enzyme, and this effect was attached to the hepatic uptake of glucose and the inhibition of gluconeogenesis generated by the action of metformin [44], which helped to reduce hyperglycemia in the animals treated with this combination. In respect to % HbA1c, the decrease in the hyperglycemic values was consistent with the HbA1c quantity; this was probably due to the correct utilization of the glucose that did not allow the production of AGEs [47]. In respect to the lipid profile, the parameters obtained after the administration of EEAc + glibenclamide indicate that this combination helps to delay the chronic incrementation of the parameters measured in comparison with the SIT2D control; however, considering the previous results, this combination is not a candidate for future investigations in our research group. In the case of the combination of EEAc + metformin, we observed a reduction in Chol and LDL-c with an increase in the LDL-c values; when the AIP was calculated, we observed a significant reduction in this value. Metformin has been described in the literature as a first-line treatment for DM type 2, being a primary pharmacological effect to control disturbed glucose metabolism; however, the completed mechanism of action of this drug is controversial due to metformin also influencing lipid/cholesterol pathways [52]. Several studies have reported the effects of metformin on atherosclerotic vascular disease in people with type 2 diabetes [52]; our results agree with the previous results presented for metformin, and in combination with EEAc in the doses administered, it is possible to achieve a correct reduction in hyperglycemia, % HbA1c, and lipid profile values, and considering the calculated AIP, the possibility of developing cardiovascular risks, vascular damage, among other mortal complications [4], can be reduced.

Finally, following the administration of OADs in combination with rutin, we observed in all the treatments a significant reduction in the hyperglycemic values during treatment. We propose that the combination of rutin with control drugs helped us achieve a significant reduction in hyperglycemia, as rutin was the only compound that was combined with the control drug, and there were also only two mechanisms of action that were involved in the reduction in hyperglycemia [25,28,43] in comparison with the combination with EEAc, which contained a higher number of compounds [21,23] that could produce other types of interactions to reduce the hyperglycemic values [14,16]. Additionally, it is important to mention that in this case, the subchronic combination of rutin with acarbose and canagliflozin did not generate the death of animals. This can be due to the possibility that in the EEAc there are more compounds [21,23] that can generate hypoglycemia, and their combination with control drugs at the doses administered in this study generated a mortal hypoglycemic effect. In respect to % HbA1c, the adequate control of hyperglycemia mediated after the subchronic administrations of rutin + acarbose, rutin + metformin, and rutin + canagliflozin were reflected in the reduction in % HbA1c. Our principal theory is that the subchronic administration of these combinations helps to reduce the concentration of glucose in the bloodstream and this reduction may help to avoid the generation of AGEs [53]. In respect to the lipid profile, the combinations of rutin + acarbosa and rutin + canagliflozin were the treatments with the greatest reductions in the parameters measured. We observed a significant reduction in Chol, Tri, and LDL-c with a significant increase in HDL-c values. Consequently, the AIPs calculated for these treatments were significantly lower than the SIT2D control; our results suggest that these combinations may help to significantly reduce the possibility of producing alterations of lipid metabolism, hypertriglyceridemia, high levels of cholesterol, and alterations between low-density lipoproteins (LDLs) and high-density lipoproteins (HDLs) [4], which are associated with an increase in mortality rates and other DM secondary complications, such as cardiovascular risk, vascular damage, diabetic nephropathy, and glaucoma, among others [34,35,36]. It is important to mention that the combination of rutin + canagliflozin was the only treatment that obtained normoglycemic values during all the treatments with the reduction in %HbA1c in all the treatments and with an important reduction in the lipid parameters measured. We propose that this combination helps to present one of the principal approaches in the management of DM, that is, the reduction in postprandial hyperglycemia in patients mediated by the prevention of hydrolysis (α-glucosidase) and the absorption of carbohydrates (SGLT-2) after food intake. The effective control of blood glucose levels being a key step in the prevention or reversion of diabetic complications, such as dyslipidemias, and other DM mortality complications, helps to improve the quality of life in diabetic patients [54].

According to the manuscript, the treatments used in this investigation showed good activity for the control of hyperglycemia, the combination of the extract obtained from the leaves of *A. cherimola* Miller, and the flavonoid rutin in combination with antidiabetic drugs showed an important improvement in the control of high blood glucose levels that is reflected in % HbA1c and the control of the lipid profile; however, further studies are necessary on the treatments with the best activity in order to determine their possible toxicity, and to reproduce our results in other experimental models to confirm the activity presented on this study.

## 4. Materials and Methods

### 4.1. Chemicals, Reagents and Drugs

Streptozocin (≥75% α-anomer basis, PN: S0130-5G), nicotinamide (≥99.5%, PN: 47865-U), sucrose (≥99.5% GC, PN: S9378-1Kg), acarbose (PN: PHR1253-500MG), canagliflozin (95%, PN: 721174-1G), glibenclamide (PN: PHR1287-1G), metformin (PN: PHR1084-500MG) were purchased from Sigma-Aldrich^®^ (Sigma^®^, Saint Louis, MO, USA). Buffer solution (citric acid/sodium hydroxide/hydrogen chloride, pH 4.00, CC: 109445) was purchased from Merck^®^ (Merck^®^, Darmstadt, Germany).

### 4.2. Plant Material

*Annona cherimola* Miller leaves were collected by Dr. Fernando Calzada in December 2019 at San Jose, Tláhuac, Mexico (19°16′32.6″ N 99°00′07.1″ W). The plant material was authenticated by Santiago Xolalpa of the Herbarium IMSSM of Mexican Institute of Social Security (IMSS) where the voucher specimen is conserved under reference number: 15,795.

### 4.3. Ethanolic Extract Preparation

The air-dried and finely powdered leaves (2.9 kg) were extracted by maceration at room temperature with EtOH (2 times × 10 L). After filtration, the extract was combined and evaporated in vacuum to yield 131 g (yield 4.5%) of green residue. The chemical characterization of rutin and other compounds was made (see Supplementary Material).

### 4.4. Animals

For the biological tests, male BALB/c strain mice 8 to 10 weeks of age (20 ± 5 g) were obtained from the Animal Facility of the Centro Médico Nacional “Siglo XXI” of the Instituto Mexicano del Seguro Social (IMSS). The mice were maintained at room temperature (22 ± 2 °C) in a natural 12-h light–dark cycle and fed with laboratory rodent diet 5001 (Lab Diet ^®^, Saint Louis, MO, USA) and water ad libitum. All research with experimental animals was conducted in accordance with the Mexican Official Norm NOM-062-ZOO-1999 [54] for Animal Experimentation and Care. All research was performed with the approval of the Hospital Ethics Committee of Specialties of the National Medical Center “Siglo XXI” of the IMSS (registration: R-2015-3601-211 and R-2019-3601-004).

### 4.5. Induction of Experimental Type 2 Diabetes

The experimental diabetes mellitus was induced according to the streptozocin-induced type 2 (SIT2D) model described by Valdes et al. [39]. Mice fasted for 16 h before receiving treatment (day 0). Streptozocin (STZ) was dissolved in a cold pH 4 buffer solution, then it was administered at 100 mg/kg intraperitoneally (IP) on days 1 and 3. Nicotinamide (NA) was dissolved in a cold saline solution and administered at 240 mg/kg IP 30 min after STZ treatment only on day 1. At the end of the treatment on day 3, a 10% sucrose solution was used ad libitum over two days. On day 5, the sucrose solution was withdrawn and substituted with water ad libitum. Then, 24 h later, the development of SIT2D was determined by measuring postprandial blood glucose levels using a conventional glucometer (ACCU-CHECK^®^ Performa Blood Glucose Systems, Roche^®^, DC, Basel, Switzerland). Additionally, to confirm the SIT2D model, β-cell function was evaluated with the administration of 5 mg/kg glibenclamide orally and measuring the decrease in glucose values 2 and 4 h after administration; according to the results, there can be confirmed the existence of functional β-cell [39,40], therefore, the generated model was classified as an experimental type 2 diabetes mellitus model.

### 4.6. Grouping

For acute and subchronic evaluations, mice were randomly divided into 14 groups (*n* = 6 each) as follows: ethanolic extract of A. cherimola (EEAc) at a dose of 300 mg/kg, rutin (Rut) 50 mg/kg, oral antidiabetic drugs metformin (Met) 850 mg/kg, glibenclamide (Gli) 5 mg/kg, acarbose (Aca) 50 mg/kg, and canagliflozin (Cana) 50 mg/kg. The combinations with EEAc were: EEAc + Met 300/850 mg/kg, EEAc + Cana (300/50 mg/kg), EEAc + Gli (300/5 mg/kg), and EEAc + Aca (300/50 mg/kg). The combinations with rutin were Rut + Aca (50/50 mg/kg), Rut + Cana (50/50 mg/kg), Rut + Met (50/850 mg/kg), and Rut+ Gli (50/5 mg/kg). All samples were dissolved in 2% Tween 80 in water as a vehicle. The normoglycemic and SIT2D control groups were treated with the vehicle (2% Tween 80 in water); all treatments were administered orally with a gavage at a volume of 0.5 mL for each animal [38].

#### 4.6.1. Acute Evaluation of Ethanolic Extract from *Annona cherimola*, Rutin, and Oral Antidiabetic Drugs on SIT2D Mice Model

Animals with blood glucose levels between 250–380 mg/dL were used for this study. The treatments described above were administered orally, once administered the treatments, the blood samples were collected from the tail vein at the beginning (0 h), 2 and 4 h after administration using a conventional glucometer (ACCU-CHECK^®^ Performa Blood Glucose Systems, Roche^®^, DC, Basel, Switzerland) [46].

#### 4.6.2. Subchronic Evaluation of Ethanolic Extract from *Annona cherimola*, Rutin, and Oral Antidiabetic Drugs on SIT2D Mice Model

Animals from the acute test continued to be used for the subchronic evaluation, all the treatments were administered daily for 8 weeks, doses are described above. Blood glucose levels were measured weekly as previously described. Additionally, lipid profile and glycated hemoglobin (HbA1c) measurement were carried out every 2 weeks.

#### 4.6.3. % HbA1c Measurement

For the measurement of glycated hemoglobin (HbA1c), blood sample were collected from the tail vein of the animals treated and were analyzed using the system automated boronate affinity assay for the determination of the percentage of Hemoglobin A1c (HbA1c%) in whole blood, Clover HbA1c reader, Infopía^®^ (Anyang, Korea).

#### 4.6.4. Lipid Profile Measurement

After eight weeks of treatment, a lipid profile was created for the study groups; to perform the measurements, blood samples were obtained from the tail vein of the animals and analyzed using VERI-Q^®^ monitoring equipment. The parameters measured were cholesterol (Chol), triglycerides (TRIGs), high-density lipoprotein cholesterol (HDL-c), low-density lipoprotein cholesterol (LDL-c), and atherogenic index of plasma (AIP).

For the atherogenic index calculation the next formula was used:

Atherogenic index of plasma (AIP)=CholHDL-c

### 4.7. Statistical Analysis

All the results are expressed as mean values ± standard error of the mean (SEM). All statistical analyses were performed using GraphPad Prism version 8.02 (GraphPad Software Inc., San Diego, CA, USA). The statistical evaluation was conducted through an analysis of variance followed by a Tukey test for multiple comparisons. *p* ≤ 0.05 was considered a statistically significant difference.

## 5. Conclusions

The complete analysis of the results performed showed the adequate management of hyperglycemia, % HbA1c, and lipid profile following the administration of ethanolic extract obtained from the leaves of *A. cherimola* and the flavonoid rutin. Additionally, for the first time, we presented the activity of ethanolic extract in combination with oral antidiabetic drugs. Our results demonstrate the importance of evaluating the activity of herbal remedies in combination with common oral drugs, as in some cases, these combinations can generate side effects in the patients. Finally, it was shown that the administration of rutin at doses of 50 mg/kg in combination with oral antidiabetic drugs helps to reduce hyperglycemia, % HbA1c, and lipid profiles, being considered as a first step to develop new treatments focusing on the control of DM and its complications.

## Figures and Tables

**Table 1:** Blood glucose levels of normoglycemic mice (NM) and streptozocin induced type 2 diabetic mice (SIT2D) at 0, 2, and 4 h, on the acute antihyperglycemic test.

Treatment	Glycemia (mg/dL)		
	0 h	2 h	4 h
NM Control	119 ± 5.2	132 ± 3.8	135 ± 4.1
SIT2D Control	385.7 ± 28.4	404.3 ± 15.3	404.7 ± 9.6
EEAc	368.3 ± 16	230 ± 15.9 *^,^^ψ^	342 ± 3.3 ^ψψ^
Rut	358 ± 7.9	323 ± 6.6 *^,^^ψ^	266.3 ± 14.4 *^,^^ψψ^
Aca	365.7 ± 15	302.6 ± 21.9 *^,^^ψ^	283.7 ± 10.3 *
Met	368 ± 14.3	388 ± 15.9 *	363.6 ± 12.6
Gli	373.7 ± 11.4	267.5 ± 30.3 *^,^^ψ^	353 ± 3.4 ^ψψ^
Cana	412.3 ± 2.2	242 ± 28.5 *^,^^ψ^	257.3 ± 46.3 *^,^^ψψ^
EEAc + Aca	350.7 ± 14.9	212.9 ± 36.7 *^,^^ψ^	219.4 ± 35.9 *^,^^ψψ^
EEAc + Met	353 ± 9.3	178.3 ± 12.7 *^,^^ψ^	156 ± 6 *^,^^ψψ^
EEAc + Glib	339.3 ± 14.2	145.7 ± 24.3 *^,^^ψ^	202 ± 42.3 *^,^^ψψ^
EEAc + Cana	348 ± 6.5	140 ± 48.5 *^,^^ψ^	135.3 ± 51 *^,^^ψψ^
Rut + Aca	340 ± 13.4	311.2 ± 19.4	250.7 ± 28.9 *^,^^ψψ^
Rut + Met	326 ± 8.4	366.7 ± 38.3	327.7 ± 34.7 ^ψψ^
Rut + Glib	316.3 ± 5.2	317.7 ± 9.1	272.4 ± 9.8 *^,^^ψψ^
Rut + Cana	337 ± 17.3	103.7 ± 9.4 *^,^^ψ^	117.7 ± 2.5 *^,^^ψψ^

Data are expressed as means ± SEM, *n* = 6; * *p* < 0.05 vs. initial values; ^ψ^ *p* < 0.05 vs. SIT2D control for 2 h; ^ψψ^ *p* < 0.05 vs. SIT2D control for 4 h SEM: standard error of the mean; NM: normoglycemic mice; SITD2: streptozocin-induced diabetes 2 mice; EEAc: ethanolic extract of *Annona cherimola* Miller; Rut: flavonoid rutin; Aca: acarbose; Met: metformin; Gli: glibenclamide; Cana: canalgiflozin.

**Table 2:** Lipid profile values of groups treated with EEAc, rutin and oral antidiabetic drugs separately.

Treatment	Parameter				
	Chol (mg/dL)	Tri (mg/dL)	HDL-c (mg/dL)	LDL-c (mg/dL)	AIP
NM Control	92 ± 1 ^♦^	88.9 ± 0.2 ^♦^	62.9 ± 0.7 ^♦^	79.4 ± 1.1	1.46 ± 0.02
SIT2D Control	163.7 ± 7.1 *	127 ± 5.5 *	29.9 ± 1 *	157.7 ± 6.9 *	5.4 ± 0.04 *
EEAc	113 ± 8.8 ^♦^	65.9 ± 4.6 ^♦^	111 ± 9.5 *^,^^♦^	90.7 ± 6.9 ^♦^	1.02 ± 0.008 *^,^^♦^
Rut	125 ± 3.3 *^,^^♦^	118 ± 8 ^♦^	91.9 ± 6.4 *^,^^♦^	107.4 ± 2 *^,^^♦^	1.3 ± 0.06 ^♦^
Aca	80.7 ± 2.1 ^♦^	201.7 ± 23.4 *^,^^♦^	78.9 ± 6.4 *^,^^♦^	64.9 ± 1.6 ^♦^	1.04 ± 0.07 ^♦^
Met	171 ± 9 *	203.7 ± 18.1 *^,^^♦^	77.9 ± 7.3 *^,^^♦^	155.4 ± 7.6 *	2.2 ± 0.09 *^,^^♦^
Gli	160 ± 4 *	87 ± 7.5 ^♦^	86 ± 6.2 *^,^^♦^	142.6 ± 5.2 *	1.8 ± 0.1 *^,^^♦^
Cana	98.9 ± 3 ^♦^	84.3 ± 2.6 ^♦^	64.9 ± 3.9 ^♦^	85.9 ± 2.2 ^♦^	1.5 ± 0.04 ^♦^

* *p* < 0.05 vs. NM Control; ^♦^ *p* < 0.05 vs. SIT2D Control.

**Table 3:** Lipid profile values of groups treated with oral antidiabetic drugs in combination with EEAc.

Treatment	Parameter				
	Chol (mg/dL)	Tri (mg/dL)	HDL-c (mg/dL)	LDL-c (mg/dL)	AIP
NM Control	92 ± 1 ^♦^	88.9 ± 0.2 ^♦^	62.9 ± 0.7 ^♦^	79.4 ± 1.1	1.46 ± 0.02
SIT2D Control	163.7 ± 7.1 *	127 ± 5.5 *	29.9 ± 1 *	157.7 ± 6.9 *	5.4 ± 0.04 *
EEAc + Met	77 ± 2.4 ^♦^	240 ± 26.8 *^,♦^	78.6 ± 4.6 ^♦^	62.2 ± 1.5 ^♦^	0.99 ± 0.02 *^,♦^
EEAc + Gli	208 ± 13.2 *^,♦^	102 ± 5.9 *^,♦^	38.9 ± 9.8 *	184 ± 11.2 *^,♦^	1.7 ± 0.03 *^,♦^

* *p* < 0.05 vs. NM Control; ^♦^ *p* < 0.05 vs. SIT2D Control.

**Table 4:** Lipid profile values of groups treated with oral antidiabetic drugs in combination with rutin.

Treatment	Parameter				
	Chol (mg/dL)	Tri (mg/dL)	HDL-c (mg/dL)	LDL-c (mg/dL)	AIP
NM Control	92 ± 1 ^♦^	88.9 ± 0.2 ^♦^	62.9 ± 0.7 ^♦^	79.4 ± 1.1	1.46 ± 0.02
SIT2D Control	163.7 ± 7.1 *	127 ± 5.5 *	29.9 ± 1 *	157.7 ± 6.9 *	5.4 ± 0.04 *
Rut + Aca	88.9 ± 1.1 **^♦^**	81.9 ± 3.8 **^♦^**	79.5 ± 3.8 *^,^**^♦^**	73 ± 0.6 **^♦^**	1.1 ± 0.04 **^♦^**
Rut + Met	163 ± 7.3 *	69.9 ± 2.1 **^♦^**	39.9 ± 3.3	155 ± 6.6 *	4.1 ± 0.1 *^,^**^♦^**
Rut + Gli	114 ± 2.3 **^♦^**	91.9 ± 5.3 **^♦^**	72.9 ± 4.1 *^,^**^♦^**	99.3 ± 1.5 **^♦^**	1.5 ± 0.05 **^♦^**
Rut + Cana	110 ± 1.7 **^♦^**	107 ± 3.9 **^♦^**	94.9 ± 6.7 *^,^**^♦^**	90.9 ± 0.3 **^♦^**	1.1 ± 0.06 **^♦^**

* *p* < 0.05 vs. NM Control; ^♦^ *p* < 0.05 vs. SIT2D Control.

## Data Availability

The data presented in this article are available on request from the corresponding authors.

## Supplementary Materials

The following supporting information can be downloaded at: https://www.mdpi.com/article/10.3390/ph16010112/s1, HPLC-DAD analysis at 254 nm of the ethanol extract of the leaves of *Annona cherimola* Miller, rutin, nicotiflorin, and narcissin; Ultraviolet spectra obtained from HPLC-DAD: rutin, nicotinflorin, and narcissin; ^1^H-NMR of rutin, nicotiflorin, and narcissin; ^13^C-NMR of rutin, nicotiflorin, and narcissin.
