# Supplementary material for: Antihyperglycemic Effects of Annona cherimola Miller and the Flavonoid Rutin in Combination with Oral Antidiabetic Drugs on Streptozocin-Induced Diabetic Mice
Source: Pharmaceuticals (Basel). 2023 Jan 12;16(1):112. doi: 10.3390/ph16010112 (PMC9865614; doi:10.3390/ph16010112)

Supplementary material

Title:
"Antihyperglycemic effects of *Annona cherimola* Miller and the flavonoid rutin in combination with oral antidiabetic drugs on streptozocin-induced diabetic mice"

Authors:
Miguel Valdes [1,*], Fernando Calzada [2,*], Jesús Martínez-Solís[1,2], Julita Martínez-Rodríguez[1,2]

Institutions:
[1] Instituto Politécnico Nacional, Sección de Estudios de Posgrado e Investigación, Escuela Superior de
Medicina, Plan de San Luis y Salvador Díaz Mirón S/N, Col. Casco de Santo Tomás, CP 11340 CDMX, Mexico
[2] UMAE Hospital de Especialidades 2◦ Piso CORSE Centro Médico Nacional Siglo XXI, Instituto Mexicano del Seguro Social, Av. Cuauhtémoc 330, Col. Doctores, CP 06720 CDMX, Mexico

Table of contents: **Page**

HPLC-DAD analysis at 254 nm of the ethanol extract of the leaves of *Annona cherimola* Miller, rutin, nicotiflorin, and narcissin — 2

Ultraviolet spectra obtained from HPLC-DAD: rutin, nicotinflorin, and narcissin — 3

^{1}H-NMR of rutin**,** nicotiflorin, and narcissin — 4

^{13}C-NMR of rutin**,** nicotiflorin, and narcissin — 5

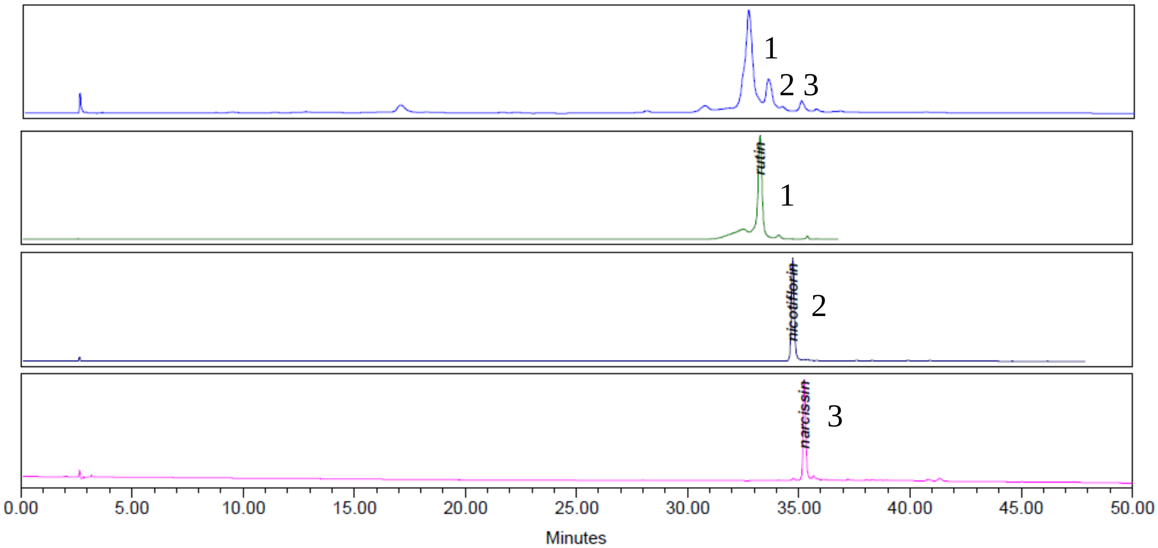

HPLC-DAD analysis at 254 nm of the ethanol extract of the leaves of *Annona cherimola* Miller (Blue), rutin (1, Green), nicotiflorin (2, Black), and narcissin (3, Pink).

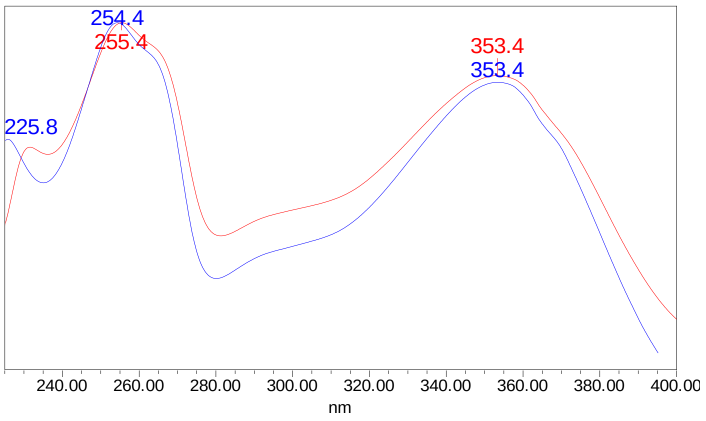

Ultraviolet spectra obtained from HPLC-DAD: rutin (red) vs standard of rutin (blue)

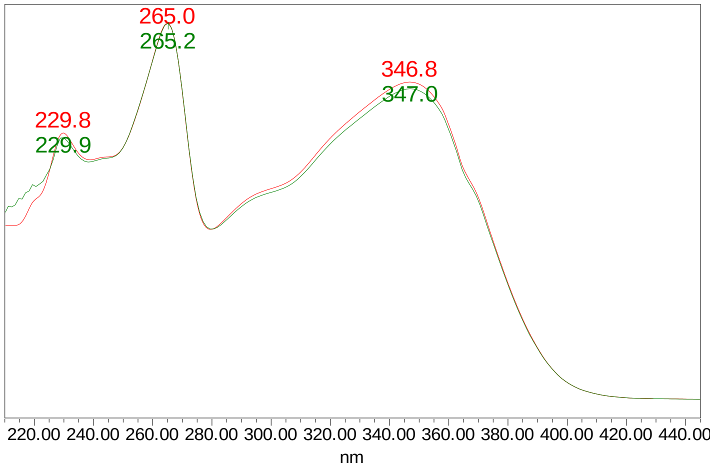

Ultraviolet spectra obtained from HPLC-DAD: nicotiflorin (red) vs standard of nicotiflorin (green)

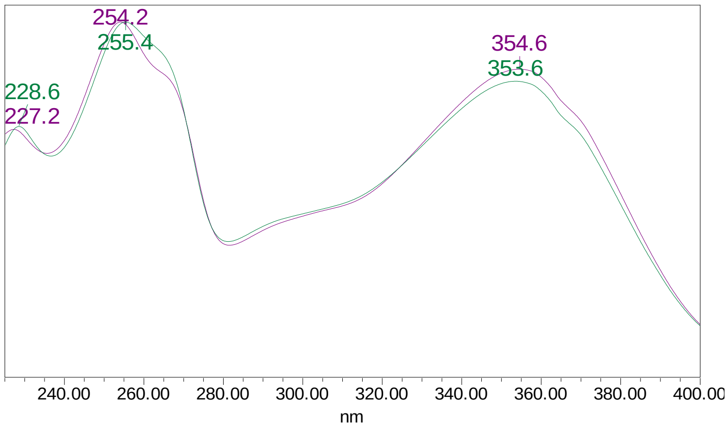

Ultraviolet spectra obtained from HPLC-DAD: narcissin (green) vs standard of narcissin (violet)

^{1}H-NMR of rutin

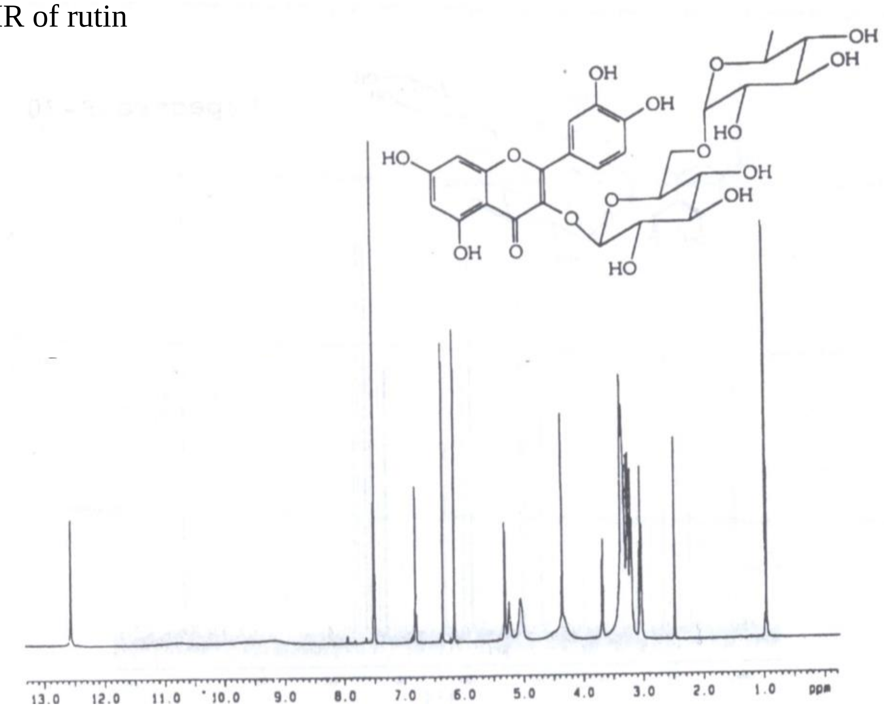

^{1}H-NMR of nicotiflorin

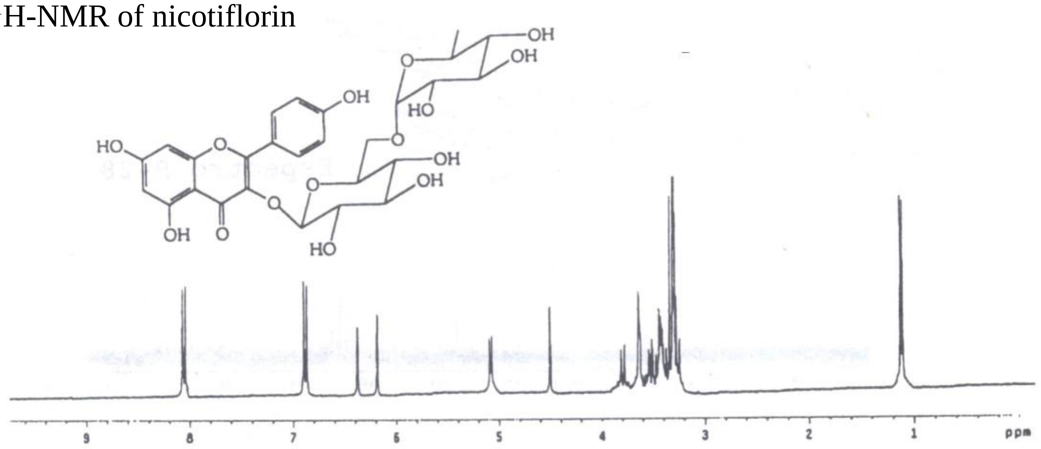

^{1}H-NMR of narcissin

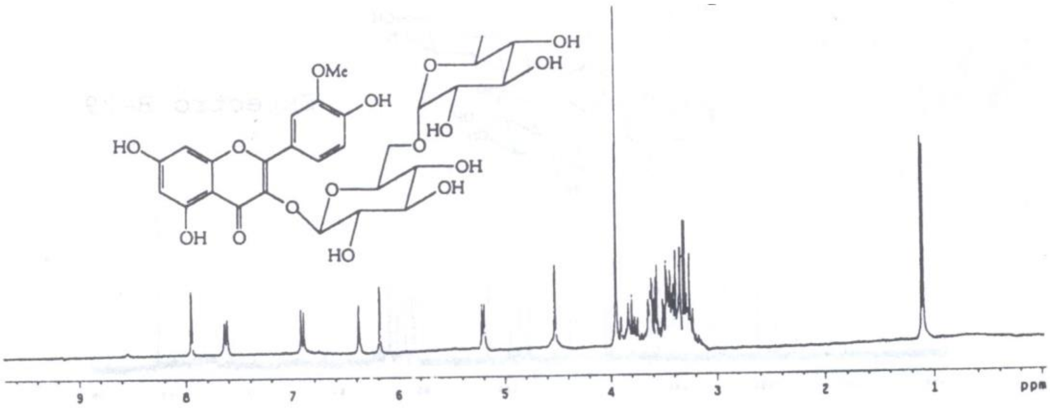

^{13}C-NMR of rutin

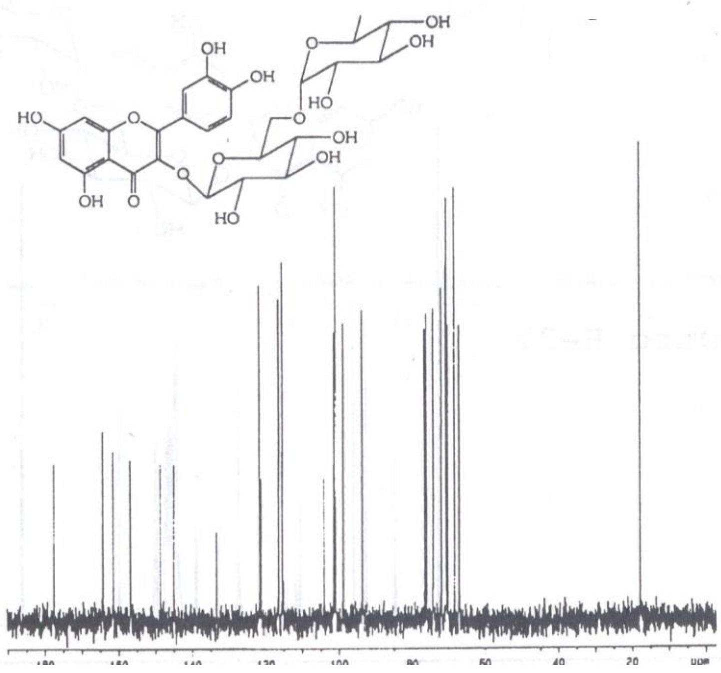

^{3}C-NMR of nicotiflorin

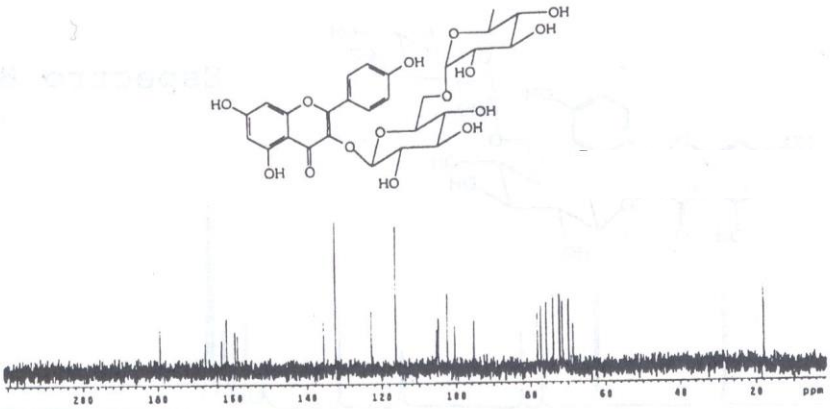

^{13}C-NMR of narcissin

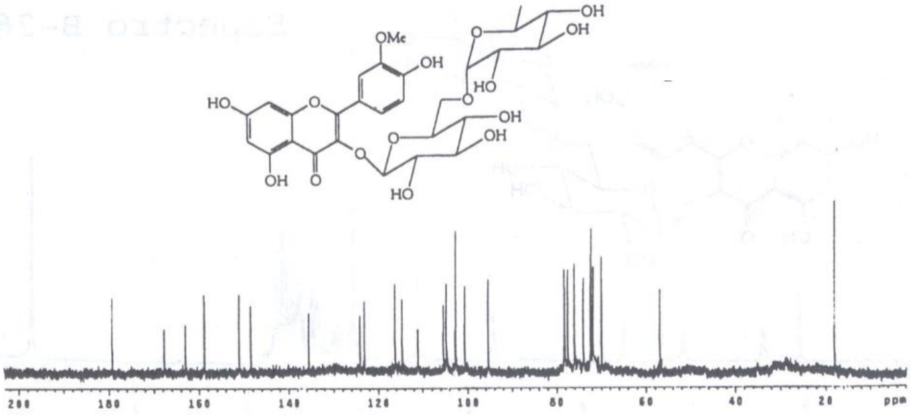

Supplement: Supplementary file 1 [file pharmaceuticals-16-00112-s001.zip › pharmaceuticals-2112438-supplementary.pdf]
